# The *CDKAL1* rs7747752-Bile Acids Interaction Increased Risk of Gestational Diabetes Mellitus: A Nested Case-Control Study

**DOI:** 10.3389/fendo.2022.808956

**Published:** 2022-03-10

**Authors:** Hui Wang, Jing Li, Junhong Leng, Weiqin Li, Jinnan Liu, Xiaoyan Yan, Zhijie Yu, Gang Hu, Ronald C. W. Ma, Zhongze Fang, Ying Wang, Xilin Yang

**Affiliations:** ^1^ Department of Epidemiology and Biostatistics, School of Public Health, Tianjin Medical University, Tianjin, China; ^2^ Tianjin Key Laboratory of Environment, Nutrition and Public Health, Tianjin Medical University, Tianjin, China; ^3^ Tianjin Center for International Collaborative Research on Environment, Nutrition and Public Health, Tianjin Medical University, Tianjin, China; ^4^ Project Office, Tianjin Women and Children’s Health Center, Tianjin, China; ^5^ School of Public Health, Shanxi Medical University, Shanxi, China; ^6^ Population Cancer Research Program and Department of Pediatrics, Dalhousie University, Halifax, NS, Canada; ^7^ Chronic Disease Epidemiology Laboratory, Pennington Biomedical Research Center, Baton Rouge, LA, United States; ^8^ Department of Medicine and Therapeutics and Li Ka Shing Institute of Health Sciences, Prince of Wales Hospital, The Chinese University of Hong Kong, Hong Kong, Hong Kong SAR, China; ^9^ Department of Toxicology and Sanitary Chemistry, School of Public Health, Tianjin Medical University, Tianjin, China; ^10^ Scientific Research Platform of the Second School of Clinical Medicine & Key Laboratory of 3D Printing Technology in Stomatology, Guangdong Medical University, Dongguan, China

**Keywords:** *CDKAL1*, rs7747752, glycoursodeoxycholic acid, deoxycholic acid, gestational diabetes mellitus

## Abstract

**Aims:**

The study aimed to explore additive interactions of *CDKAL1* rs7747752 and GUDCA/DCA for GDM risk and whether the interactive effects on the risk of GDM was mediated *via* increasing lysophosphatidylcholines (LPC) 18:0 and/or saturated fatty acid (SFA) 16:0.

**Methods:**

A 1:1 age-matched study nested in a prospective cohort of pregnant women (207 pairs) was organized in Tianjin, China. Additive interactions were used to test interaction effects while mediation analyses and Sobel tests were used to test mediation effects of LPC18:0 and SFA16:0 between copresence of rs7747752 and low GUDCA/DCA, and GDM risk.

**Results:**

The *CDKAL1* rs7747752 was associated with GDM (P<0.05). The rs7747752 C polymorphism markedly enhanced ORs of low GUDCA from 4.04 (0.72-22.8) to 9.02 (1.63-49.7) and low DCA from 1.67 (0.68-4.11) to 4.24 (1.84-9.76), both with significant additive interactions. Further adjustment for LPC18:0 attenuated the interactive effects of rs7747752 and low DCA, with a significant mediation effect (P=0.003). High SFA16:0 did not mediate the interactive effects of rs7747752 and low DCA/GUDCA on GDM risk.

**Conclusions:**

The *CDKAL1* rs7747752 C carrier status and low GUDCA/DCA had significant additive interactions on the risk of GDM with the effect from interaction with DCA being partially mediated *via* increasing LPC18:0.

## Introduction

The prevalence of gestational diabetes mellitus (GDM) has been rapidly increasing worldwide (1). GDM is not only associated with perinatal adverse outcomes but also predispose women to increased risk of diabetes and cardiovascular disease in later life, and their offspring to increased risk of childhood obesity (2, 3). On the other hand, intensive management of GDM does not have a detectable effect on the risk of postpartum diabetes in the mothers (4) and childhood obesity in their offspring (5). How to prevent GDM has become increasingly important to reduce the burden of GDM. However, our meta-analysis of 29 randomized controlled trials shows that lifestyle interventions within 15 weeks of pregnancy can only achieve as low as 20% reduction in risk of GDM while later lifestyle interventions are ineffective in reducing the risk (6). Indeed, a better understanding of the pathophysiology of GDM and identification of its novel biomarkers are critically important for the prediction and prevention of GDM in early pregnancy.

GDM is a complex disease determined by a constellation of factors, including genetic, metabolic, and other environmental factors (7). Among genetic factors, single nucleotide polymorphisms (SNP) within the cyclin-dependent kinase 5 regulatory subunit-associated protein1-like 1 (*CDKAL1*) locus was found to be strongly associated with GDM by genome-wide association analysis (GWAS) and dozens of replication studies in different populations (8–10). *CDKAL1* gene is located on the short arm of human chromosome 6 and encodes a protein of 579 amino acids, which is indeed a member of the methylthiotransferase that specifically modifies transfer ribonucleic acid (tRNA) Lys in mammals (11). The *CDKAL1* gene plays a role in the cell cycle control of beta cells by inhibiting the activity of cyclin-dependent kinase 5 (CDK5) and acting as a tRNA-modifying enzyme (12). The association between *CDKAL1* rs7747752 polymorphism and GDM was also reported in Chinese pregnant women (8).

In addition to genetic origin and environmental factors, various metabolic factors, such as bile acids (BAs) and saturated fatty acids (SFAs), can act as risk factors (13, 14). In this connection, our group found that serum glycoursodeoxycholic acid (GUDCA) ≤ 0.07 nmol/mL and deoxycholic acid (DCA) ≤ 0.28 nmol/mL in early pregnancy were independently associated with markedly increased risk of GDM in Chinese pregnant women (15). Interestingly, adjustment for LPC18:0 attenuated the risk associations of both low DCA and low GUDCA with GDM while low DCA and low GUDCA enhanced the risk association between LPC18:0 and GDM (16). The interaction between genetic disposition and metabolic factors plays a critical role in the development of GDM (17). Indeed, the CDKAL1 gene was related to defects in the conversion of proinsulin (18) and insulin response under glucose stimulation (19, 20). It is known that defection of insulin response reduces its ability to inhibit lipolysis in adipose tissue, further resulting in increased flow of SFAs into the circulation (21). In line with these findings from mechanistic investigations, our group reported that high SFA16:0 played a vital role in the risk of GDM, which had a significant interactive effect with *CDKAL1* rs7747752 on the risk of GDM (14). In addition, an animal study showed that defection of *CDKAL1* gene led to impaired insulin secretion and longitudinal fluctuations in insulin sensitivity during high-fat feeding in mice (22). Notably, BAs, as cholesterol-derived metabolites, have a well-established role in the digestion and absorption of dietary fats (23); furthermore, BAs also affect insulin secretion (24, 25). This observation suggests that BAs and *CDKAL1* variants may have a synergistic effect on the risk of GDM, possibly being mediated by LPC18:0 or SFA16:0. Indeed, it is worthwhile to explore whether abnormal BAs have any interactions with *CDKAL1* gene variants towards increased risk of GDM. It is also quite interesting to understand potential interrelationships among *CDKAL1*, BAs, SFAs, and LPCs and their roles in the etiology of GDM.

Using an age-matched case-control study nested in a large population-based cohort of pregnant women in Tianjin, China, this analysis aimed to explore 1) additive interactions between *CDKAL1* rs7747752 polymorphism and low GUDCA/DCA for the risk of GDM; and 2) whether the additive interactive effect if any between rs7747752 polymorphism and low GUDCA/DCA on the risk of GDM was mediated *via* LPC18:0 and/or SFA16:0.

## Materials and Methods

### Research Design and Population

The design and method of this study have been described previously (26). To be brief, we set up a prospective cohort study of 22 302 pregnant women at their first antenatal care with median 10th gestational weeks in Tianjin, China, from October 2010 to August 2012. The ethics of the study protocol was approved by the Ethics Committee of Tianjin Women and Children’s Health Center (TWCHC), and written informed consent was obtained before data collection.

A two-step screening procedure was used to identify GDM. First, a 1-h 50-g glucose challenge test (GCT) was performed on pregnant women at 24-28^th^ gestational weeks in the primary hospital. Second, a 2-hour 75-g oral glucose tolerance test (OGTT) was performed on pregnant women with GCT ≥ 7.8 mmol/L at the GDM clinic in TWCHC. The diagnosis of GDM is based on the cutoff points of the International Association of Diabetes and Pregnancy Study Group (27). Of the 22 302 participants, 2 991 pregnant women donated fasting blood samples. We finally included 207 GDM cases and 207 non-GDM controls who were matched by maternal age (± 1 year) (15). Data from the 207 pairs of women with high-quality SNP (28) were used to explore the research questions of this study. The flowchart of the study participants was available elsewhere (14).

### Data Collection Procedures

Data were collected during the first antenatal care visit, GCT, OGTT, and the postpartum period, which has been described in detail (26). Demographic, clinical and lifestyle information included age, weight, height, systolic/diastolic blood pressure (SBP/DBP), ethnicity, education level, parity, family history of diabetes, in first degree relatives, smoking and drinking habits before and during pregnancy.

### Measurement of Serum BAs, LPCs and SFAs

Liquid chromatography-tandem mass spectrometry (LC–MS/MS) was used to assay the concentrations of serum BAs, LPCs, and SFAs. The detailed measurement methods of serum BAs, LPCs, and SFAs were available elsewhere (14–16).

### Genotyping

Genotyping was conducted using the Illumina Infinium^®^ Global Screening Array and genotype data was imputed using minimal 3 with the 1000 Genomes Project phase 3 v 5 as reference panel. Genotyping data from specific candidate SNP (rs7747752) were extracted from the genome-wide genotyping data. The overall genotype call rate was 99.4%.

### Statistical Analysis

Power and sample size analysis was performed using PASS 15 (NCSS, LLC. Kaysville, Utah, USA). In the 1:1 age-matched case-control study, we assumed that the probability of exposure among sampled control patients is 10% and the correlation coefficient for exposure between matched case and control patents is 0.2, and type I error is set at 0.05. The sample size required to achieve 85% power for an odds ratio (OR) of 2.0 is 256 (128 GDM and 128 control). Therefore, the current sample size of our study (n=414) had more than 85% power to detect the assumed risk association.

All other statistical analyses were performed using Statistical Analysis System (SAS) release 9.4 (SAS Institute, Cary, NC). Quantitative data were compared between the GDM group and the non-GDM group, using the paired Student’s t-test or Wilcoxon signed-rank test. The categorical data were compared using the McNemar test or Fisher’s exact test. In addition, Spearman correlation analysis was used to calculate the correlation coefficients of these serum BAs and rs7747752 with neonatal birth weight. In addition, Kruskal-Wallis test was used to test the differences of serum levels of BAs between different rs7747752 genotypes. In this analysis, a P-value < 0.05 was regarded to be statistically significant.

In our previous analysis, we detected that GUDCA ≤ 0.07 nmol/mL, DCA ≤ 0.28 nmol/mL, LPC18:0 ≥ 18.0 nmol/mL and SFA16:0 ≥ 17.1 nmol/mL were independently associated with markedly increased risk of GDM in restricted cubic spline analysis (14–16). In this analysis, we used the same cutoff points of GUDCA, DCA, LPC18:0 and SFA16:0 to define low GUDCA, low DCA, high LPC18:0 and high SFA16:0. Conditional logistic regressions were used to obtain the ORs and their 95% confidence intervals (CIs) of rs7747752 and GUDCA/DCA for the risk of GDM in unadjusted models and adjusted models. The adjusted models considered confounding effects of traditional risk factors of GDM, including pre-pregnancy body mass index (BMI), family history of diabetes in first degree relatives, SBP, current smoker before pregnancy, and weight gain to the time of GCT. Next, the combined effects of rs7747752 genotypes (CC vs. CG vs. GG) and low serum levels of GUDCA/DCA on GDM susceptibility were examined to explore whether the rs7747752 and low GUDCA/DCA have potential interactive effects on the risk of GDM. Then, based on the results of combined effects of rs7747752 genotypes (CC vs. CG vs. GG) and low GUDCA/DCA on GDM, we further tested the additive interactions between rs7747752 C risk allele and low GUDCA/DCA for GDM. Three measures, i.e., relative excess risk due to the interaction (RERI), attributable proportion due to the interaction (AP), and synergy index (SI) were used to judge additive interactions (29). Any of RERI > 0, AP > 0 or SI > 1 indicates a significant additive interaction.

In order to shed light on the potential mechanisms of the additive interactions between CDKAL1 rs7747752 C allele and low GUDCA/DCA on the risk of GDM, we further conducted mediation analyses to examine whether high LPC18:0 and/or SFA16:0 can account for the association between copresence of CDKAL1 rs7747752 C allele carrier status and low GUDCA/DCA, and the increased risk of GDM. First, we estimated the ORs of copresence of rs7747752 and low GUDCA/DCA for high LPC18:0 and high SFA16:0, and the ORs of high LPC18:0 and high SFA16:0 for GDM. Then, we calculated the ORs of copresence of rs7747752 and low GUDCA/DCA for GDM after adjusting for high LPC18:0 and high SFA16:0. At last, Sobel test was used to assess the mediation effects of LPC18:0 and SFA16:0 (30).

## Results

### Characteristics of the Research Participants

The clinical and biochemical information of the study participants was shown in **Table 1**. The mean age of participants at the first antenatal care visit was 29.24 ± 3.04 standard deviation (SD) years. There were no differences in height, ethnicity, education level, parity, weight gain to GCT, smoking and drinking habits before and during pregnancy among the controls and GDM groups (P > 0.05). Women with GDM had significantly higher weight, BMI, SBP, DBP and GCT glucose levels than the controls (P < 0.05). Women with GDM also had a higher proportion of family history of diabetes in first degree relatives than the non-GDM group. Women with GDM had a lower serum level of GUDCA and DCA while had a high serum level of LPC18:0 and SFA16:0, compared with non-GDM women. The frequencies of the heterozygote (CG) and homozygous (CC) of the *CDKAL1* rs7747752 were found to be significantly higher in women with GDM than in the controls (non-GDM) (P < 0.05). Whereas, we failed to find any significant differences of serum levels of GUDCA and DCA between rs7747752 genotypes (**Table S1**).

**Table 1:** Clinical and biochemical characteristics of GDM and non-GDM women.

Characteristic	Non-GDM (n = 207)	GDM (n = 207)	*P* value
**Variables at registration**			
Age, years	29.23 ± 3.34	29.25 ± 2.74	0.480*
Height, cm	162.98 ± 4.54	163.25 ± 5.04	0.509*
Weight, kg	58.58 ± 9.78	63.87 ± 10.54	<0.001*
BMI, kg/m^2^	22.04 ± 3.46	23.95 ± 3.66	<0.001*
Systolic blood pressure, mmHg	104.21 ± 10.60	108.21 ± 10.54	<0.001*
Diastolic blood pressure, mmHg	67.91 ± 7.66	70.72 ± 7.93	<0.001*
Han ethnicity	200 (96.62)	202 (97.58)	0.564**
Education > 12 years	113 (54.59)	109 (52.66)	0.683**
Parity ≥ 1	10 (4.83)	13 (6.28)	0.532**
Family history of diabetes in first degree relatives	13 (6.28)	26 (12.56)	0.033**
Current smoker before pregnancy	13 (6.28)	14 (6.76)	0.841**
Alcohol drinker before pregnancy	52 (25.12)	63 (30.43)	0.564**
**Variables during pregnancy**			
Current smoker during pregnancy	1 (0.48)	2 (0.97)	0.564**
Alcohol drinker during pregnancy	2 (0.97)	2 (0.97)	1.000**
gestational weeks at GCT, week	25.16 ± 2.28	24.95 ± 1.44	0.033*
Weight gain to GCT, kg/week	0.58 ± 0.21	0.56 ± 0.23	0.532*
GCT glucose, mmol/L	6.39 ± 1.35	9.30 ± 1.45	<0.001*
**Bile acid species**			
GUDCA ≤ 0.07 nmol/mL	167 (80.68)	199 (96.14)	<0.001**
DCA ≤ 0.28 nmol/mL	111 (53.62)	139 (67.15)	0.006**
**Lysophosphatidylcholines species**			
LPC18:0 ≥ 18.0 nmol/mL	40 (19.32)	175 (84.54)	<0.001**
**Saturated fatty acids species**			
SFA16:0 ≥ 17.1 nmol/mL	87 (42.03)	125 (60.39)	<0.001**
***CDKAL1* gene**			
rs7747752 (C/G)			0.003**
GG	64 (30.92)	43 (20.77)	
CG	101 (48.79)	103 (49.76)	
CC	42 (20.29)	61 (29.47)	

GDM, gestational diabetes mellitus; BMI, body mass index; GCT, glucose challenge test; GUDCA, glycoursodeoxycholic acid; DCA, deoxycholic acid; LPC, Lysophosphatidylcholines. SFA, Saturated fatty acid; CDKAL1, cyclin-dependent kinase 5 regulatory subunit-associated protein1-like 1.

Data are reported in mean ± SD or number (percentages).

*Derived from paired t-test or Wilcoxon signed-rank test.

**Derived from McNemar test or Fisher’s exact test.

In addition, Spearman correlation analysis was used to calculate the correlation coefficients of these serum BAs and rs7747752 with neonatal birth weight. The correlation coefficients of DCA, GUDCA and rs7747752 with birth weight were 0.063 (P = 0.199), -0.022 (P = 0.658) and -0.017 (P = 0.728), respectively, all being not significant.

### Associations of *CDKAL1* rs7747752 and Low Serum Levels of GUDCA/DCA With GDM

The GUDCA ≤ 0.07 nmol/mL was associated with increased risk of GDM (OR: 5.84, 95% CI: 2.13-16.0) after adjustment for traditional risk factors, including pre-pregnancy BMI, family history of diabetes in first degree relatives, SBP, current smoker before pregnancy and weight gain to the time of GCT. Similarly, after adjustment for traditional risk factors, DCA ≤ 0.28 nmol/mL was also associated with increased risk of GDM (OR: 2.28, 95% CI: 1.43-3.64). In the cohort, the C allele frequency for rs7747752 was 49.89%. The *CDKAL1* rs7747752 C allele was significantly associated with GDM in the unadjusted model (OR: 1.48, 95% CI: 1.12-1.96) and adjusted model (OR: 1.76, 95% CI: 1.26-2.44). After adjustment for traditional risk factors, heterozygote (CG) and homozygote (CC) genotypes of *CDKAL1* rs7747752 were all associated with increased risk of GDM with ORs being 1.95 (95% CI: 1.15-3.31) and 3.07 (95% CI: 1.59-5.95), respectively. In dominant and recessive models, the ORs of the genotype distribution of rs7747752 in the adjusted model were 2.19 (95% CI: 1.31-3.65) and 1.88 (95% CI: 1.13-3.15), respectively (**Table 2**).

**Table 2:** Odds ratios of the *CDKAL1* rs7747752 and bile acid metabolisms for increased risk of gestational diabetes mellitus.

	Unadjusted Model		Adjusted Model	
	OR (95% CI)	*P* value	OR (95% CI)	*P* value
**Metabolites**				
GUDCA ≤ vs. > 0.07 nmol/mL	7.40 (2.91-18.8)	<0.001	5.84 (2.13-16.0)	<0.001
DCA ≤ vs. > 0.28 nmol/mL	1.74 (1.17-2.59)	0.007	2.28 (1.43-3.64)	<0.001
**Genetic variants**				
rs7747752				
GG	1.00	–	1.00	–
CG	1.51 (0.95-2.41)	0.080	1.95 (1.15-3.31)	0.014
CC	2.19 (1.24-3.85)	0.007	3.07 (1.59-5.95)	0.001
Additive (CC vs. CG vs. GG)	1.48 (1.12-1.96)	0.007	1.76 (1.26-2.44)	0.001
Dominant (CC/CG vs. GG)	1.68 (1.08-2.62)	0.023	2.19 (1.31-3.65)	0.003
Recessive (CC vs. CG/GG)	1.63 (1.04-2.57)	0.034	1.88 (1.13-3.15)	0.016

OR, odds ratios; CI, confidence intervals; GUDCA, glycoursodeoxycholic acid; DCA, deoxycholic acid.

Adjusted Model, adjusted for traditional risk factors, including pre-pregnancy body mass index, family history of diabetes in first degree relatives, systolic blood pressure, current smoker before pregnancy and weight gain to the time of glucose challenge test.

### Combined Effects With rs7747752 Genotypes (CC vs. CG vs. GG) and Low Serum Levels of GUDCA/DCA for the Risk of GDM

Using the GG genotype of rs7747752 and serum GUDCA > 0.07 nmol/mL as the reference, GUDCA ≤ 0.07 nmol/mL combined with CG and CC were associated with significantly increased risk of GDM after adjustment for traditional GDM risk factors, and the ORs were 9.58 (95% CI: 1.60-57.5) and 16.5 (95% CI: 2.59-106), respectively. In the same vein, using the GG genotype of rs7747752 and DCA > 0.28 nmol/mL as the reference, the combinations of rs7747752 CG or CC genotypes and DCA ≤ 0.28 nmol/mL were associated with markedly increased risk of GDM and ORs were 3.51 (95% CI: 1.50-8.24) and 8.06 (95% CI: 2.85-22.8), respectively (**Table 3**).

**Table 3:** Risk associations of combinations of rs7747752 genotypes (CC vs. CG vs. GG) and low serum levels of GUDCA/DCA with gestational diabetes mellitus.

Genetic variants	Metabolites	Non-GDM (n=207)	GDM (n=207)	Unadjusted Model		Adjusted Model	
				OR (95% CI)	*P* value	OR (95% CI)	*P* value
**Combinations of rs7747752 genotypes and GUDCA**							
rs7747752	GUDCA (in nmol/mL)						
GG	>0.07	10 (4.83)	2 (0.96)	1.00	–	1.00	–
GG	≤0.07	54 (26.09)	41 (19.81)	6.32 (1.07-37.2)	0.042	4.88 (0.80-29.8)	0.086
CG	>0.07	22 (10.63)	5 (2.42)	1.81 (0.26-12.4)	0.546	2.42 (0.34-17.3)	0.378
CG	≤0.07	79 (38.16)	98 (47.34)	10.4 (1.79-60.5)	0.009	9.58 (1.60-57.5)	0.013
CC	>0.07	8 (3.86)	1 (0.48)	0.54 (0.04-6.74)	0.634	0.48 (0.03-6.95)	0.589
CC	≤0.07	34 (16.43)	60 (28.99)	15.9 (2.61-97.1)	0.003	16.5 (2.59-106)	0.003
**Combinations of rs7747752 genotypes and DCA**							
rs7747752	DCA (in nmol/mL)						
GG	>0.28	27 (13.04)	15 (7.25)	1.00	–	1.00	–
GG	≤0.28	37 (17.87)	28 (13.53)	1.40 (0.63-3.09)	0.406	1.70 (0.69-4.22)	0.250
CG	>0.28	44 (21.26)	34 (16.43)	1.38 (0.61-3.09)	0.435	1.57 (0.62-3.95)	0.341
CG	≤0.28	57 (27.54)	69 (33.33)	2.13 (1.03-4.38)	0.040	3.51 (1.50-8.24)	0.004
CC	>0.28	25 (12.08)	19 (9.18)	1.43 (0.58-3.53)	0.436	2.03 (0.71-5.75)	0.185
CC	≤0.28	17 (8.21)	42 (20.29)	4.66 (1.90-11.4)	<0.001	8.06 (2.85-22.8)	<0.001

OR, odds ratios; CI, confidence intervals; GUDCA, glycoursodeoxycholic acid; DCA, deoxycholic acid.

Adjusted Model, adjusted for traditional risk factors, including pre-pregnancy body mass index, family history of diabetes in first degree relatives, systolic blood pressure, current smoker before pregnancy and weight gain to the time of glucose challenge test.

### Additive Interactions Between rs7747752 C Allele (C vs. G) and Low Serum Levels of GUDCA/DCA for the Risk of GDM

Using rs7747752 G allele and GUDCA > 0.07 nmol/mL as the reference, the presence of rs7747752 C allele markedly increased the OR of GUDCA ≤ 0.07 nmol/mL for GDM in adjusted model from 4.04 (95% CI: 0.72-22.8) for GUDCA ≤ 0.07 nmol/mL alone to 9.02 (95% CI: 1.63-49.7) for the presence of both. There was a significant additive interaction between *CDKAL1* rs7747752 C allele and GUDCA ≤ 0.07 nmol/L for GDM (AP: 0.50, 95% CI: 0.17-0.83).

Similarly, *CDKAL1* rs7747752 C allele also markedly increased OR of DCA ≤ 0.28 nmol/mL for GDM in adjusted model from 1.67 (95% CI: 0.68-4.11) for DCA ≤ 0.28 nmol/mL alone to 4.24 (95% CI: 1.84-9.76) for copresence of both. The additive interaction was also significant (AP: 0.46, 95%CI: 0.09-0.83) (**Table 4**).

**Table 4:** Additive interactions between rs7747752 C allele (C vs. G) and low GUDCA/DCA for the risk of gestational diabetes mellitus.

	Unadjusted Model		Adjusted Model	
	OR/Estimate	*P* value	OR/Estimate	*P* value
	(95% CI)		(95% CI)	
**Additive interaction between rs7747752 risk allele C (vs. G) and low GUDCA**				
rs7747752 G allele & GUDCA > 0.07 nmol/mL	1.00	–	1.00	–
rs7747752 G allele & GUDCA ≤ 0.07 nmol/mL	5.52 (1.02-29.8)	0.047	4.04 (0.72-22.8)	0.113
rs7747752 C allele & GUDCA > 0.07 nmol/mL	1.20 (0.21-6.95)	0.838	1.43 (0.23-8.93)	0.700
rs7747752 C allele & GUDCA ≤ 0.07 nmol/mL	10.1 (1.90-53.5)	0.007	9.02 (1.63-49.7)	0.012
RERI	4.36 (-2.53-11.3)		4.54 (-2.66-11.7)	
AP	0.43 (0.11-0.76)		0.50 (0.17-0.83)	
SI	1.92 (0.90-4.13)		2.31 (0.85-6.24)	
**Additive interaction between rs7747752 risk allele C (vs. G) and low DCA**				
rs7747752 G allele & DCA > 0.28 nmol/mL	1.00	–	1.00	–
rs7747752 G allele & DCA ≤ 0.28 nmol/mL	1.36 (0.62-2.99)	0.444	1.67 (0.68-4.11)	0.266
rs7747752 C allele & DCA > 0.28 nmol/mL	1.34 (0.63-2.88)	0.448	1.64 (0.68-3.93)	0.269
rs7747752 C allele & DCA ≤ 0.28 nmol/mL	2.55 (1.26-5.17)	0.009	4.24 (1.84-9.76)	<0.001
RERI	0.85 (-0.27-1.96)		1.93 (-0.09-3.96)	
AP	0.33 (-0.12-0.79)		0.46 (0.09-0.83)	
SI	2.20 (0.33-14.8)		2.48 (0.66-9.34)	

OR, odds ratios; CI, confidence intervals; GUDCA, glycoursodeoxycholic acid; DCA, deoxycholic acid; RERI, relative excess risk due to interaction; AP, attributable proportion due to interaction; SI, synergy index.

Adjusted model, adjusted for traditional risk factors, including pre-pregnancy body mass index, family history of diabetes in first degree relatives, systolic blood pressure, current smoker before pregnancy and weight gain to the time of glucose challenge test.

### Mediation Effects of LPC18:0 and SFA16:0 for Copresence of rs7747752 C Allele (C vs. G) and Low Serum Levels of GUDCA/DCA to the Risk of GDM

In adjusted model analysis, copresence of rs7747752 C allele and DCA ≤ 0.28 nmol/mL was associated with markedly increased risk of high LPC18:0 (OR: 4.51, 95% CI: 1.79-11.3). Adjustment for LPC18:0 greatly attenuated the OR of copresence of rs7747752 C allele and DCA ≤ 0.28 nmol/mL from 4.24 (1.84-9.76) to 2.67 (0.71-9.99). The mediation effect of LPC18:0 on the risk association of copresence of rs7747752 C risk allele and DCA ≤ 0.28 nmol/mL with GDM was statistically significant (P of Sobel test: 0.003) (**Table 5**). On the other hand, the mediation effect of LPC18:0 on the risk association of rs7747752 C risk allele and GUDCA ≤ 0.07 nmol/mL with GDM was non-significant (P of Sobel test: 0.114). Whereas, further adjustment for SFA16:0 markedly enhanced the interactive effects of rs7747752 C risk allele and low GUDCA on the risk of GDM up to 7.20 (95% CI: 0.87-59.4), and slightly enhanced the risk association of rs7747752 C risk allele and low DCA with GDM (OR: 4.36, 95% CI: 1.83-10.4), both without any mediation effects (**Table S2**).

**Table 5:** Mediation effect of LPC18:0 for interaction between rs7747752 C allele (C vs. G) and low DCA to increased risk of gestational diabetes mellitus.

	Beta (SD)	OR (95% CI)	*P* value
**Model A (LPC18:0 ≥ 18.0 nmol/mL as the outcome)**			
rs7747752 C allele & DCA ≤ 0.28 nmol/mL	1.51 (0.47)	4.51 (1.79-11.3)	0.001
**Model B (GDM as the outcome)**			
LPC18:0 ≥ vs. < 18.0 nmol/mL	2.89 (0.38)	18.1 (8.51-38.3)	<0.001
**Model C (GDM as the outcome)**			
rs7747752 C allele & DCA ≤ 0.28 nmol/mL	0.98 (0.67)	2.67 (0.71-9.99)	0.145
**Sobel test for mediation effect** ^†^			0.003

SD, standard definition; OR, odds ratios; CI, confidence intervals; LPC, lysophosphatidylcholines; GDM, gestational diabetes mellitus; DCA, deoxycholic acid.

Model A was adjusted for the variables listed in the adjusted model in **Table 4**.

Model B was adjusted for traditional risk factors in the adjusted model in **Table 4**.

Model C was further adjusted for LPC18:0 ≥ 18.0 nmol/mL in addition to the variables listed in the adjusted model in **Table 4**.

^†^P value of Sobel test < 0.05 indicating significant mediation effect.

## Discussion

Our study found significant additive interactions between *CDKAL1* rs7747752 C allele carrier status and low serum levels of GUDCA and DCA for the markedly increased risk of GDM in Chinese pregnant women. The additive interactive effect of *CDKAL1* rs7747752 C allele carrier status and low serum levels of DCA on GDM was partially mediated *via* high LPC18:0. However, we failed to confirm that the copresence of *CDKAL1* rs7747752 C allele carrier status and low GUDCA on the risk of GDM was also mediated *via* high LPC18:0. In addition, the additive interactions between *CDKAL1* rs7747752 C allele carrier status and low serum levels of GUDCA and DCA on GDM risk were independent of high SFA16:0.

The role of BAs in the regulation of glucose metabolism is a hot topic of recent diabetes research. Several studies in pregnant women have attempted to address associations between BAs and GDM with inconsistent findings. A nested case-control study of 131 women with GDM and 138 controls in early pregnancy did not find significant differences in DCA, ursodeoxycholic acid (UDCA), and GUDCA between GDM and non-GDM women, but did observe that the primary BAs were down-regulated while some other secondary BAs were up-regulated in GDM versus non-GDM in the univariate analysis (31). Two other studies also did not detect a significant difference in DCA and UDCA between GDM women and their control group at 28^th^ gestational weeks (13, 32). Using the restricted cubic spline technique, our group found that GUDCA and DCA had clear threshold effects on the risk of GDM, with the GUDCA ≤ 0.07 nmol/mL and the DCA ≤ 0.28 nmol/mL being associated with markedly increased risk of GDM (15). Our findings further indicated that women in low serum levels of GUDCA and DCA with rs7747752 C risk alleles had markedly increased the risk of GDM. It is worthwhile to investigate whether population differences in proportions of *CDKAL1* rs7747752 C allele carriers contributed to the inconsistent findings regarding the association between BA metabolites and the risk of GDM in different studies.

*CDKAL1* gene has a strong association with GDM among the genes identified to date. *CDKAL1* genetic variants also have been shown to predict GDM and related glycemic traits (10), suggesting that it might have a synergistic effect with other risk factors. The rs7747752 under study was significantly associated with increased GDM risk. Similar to our findings, another study also found that Chinese women with the rs7747752 polymorphism were predisposed to a high risk of GDM (8). Previous studies have reported significant interaction effects of *CDKAL1* with lifestyle interventions, SFAs, and vitamin D on GDM risk (14, 17, 33). In a recent study, our group found that *CDKAL1* genetic variants had a significant additive interaction with serum SFAs, resulting in a markedly increased risk of GDM (14). The interaction between both risk factors may suggest that GDM develops when impaired beta-cell function cannot produce enough insulin in response to increased insulin resistance as manifested by high SFA16:0. In this analysis, we further found that the *CDKAL1* rs7747752 C allele carrier status had significant additive interactions with low GUDCA and low DCA for the risk of GDM, independent of high SFA16:0. Interestingly, LPC18:0 mediated the interactive effect of *CDKAL1* rs7747752 and low DCA on the risk of GDM.

To our knowledge, our study was the first that investigated additive interactions between the *CDKAL1* genetic variants (rs7747752) and low serum levels of GUDCA/DCA for the risk of GDM. We also observed that LPC18:0 but not SFA16:0 mediated the interactive effects of rs7747752 and low DCA on the risk of GDM. The functions of *CDKAL1* and methylthiotransferase in the regulation of metabolism are not fully understood, so do the mechanisms underlying the additive interactions between *CDKAL1* rs7747752 and low GUDCA/DCA. Recent studies have suggested several biological links between BAs and glucose regulation. First, a biological link between decreased DCA/GUDCA and GDM is plausible. Some BAs can activate FXR in the intestine stimulate the synthesis of fibroblast growth factor (FGF) 15/19 (34) and upregulate expression of pancreatic beta cells (35), which exerts pleiotropic effects on hepatic BAs metabolism as well as lipid, protein, and glucose metabolism (34). What’s more, BAs-mediated TGR-5 signaling increases the release of GLP-1, which augments glucose-stimulated insulin secretion from pancreatic beta cells (36). Notably, the *CDKAL1* gene is tightly associated with impaired beta-cell function (37) and insulin secretion (38), thus being associated with increased risk of GDM. Hence, it is possible that copresence of low GUDCA/DCA and *CDKAL1* genetic variants markedly increased the risk of GDM *via* impaired beta-cell function. Second, multiple lines of evidence support a pathway from the additive interactions of low DCA/GUDCA and *CDKAL1* to increased LPC18:0 could play a role in the etiology of GDM. A mouse study found that increased intestinal taurine-beta-muocholic acid, an antagonist of FXR, reduced high-fat diet-induced increase in LPCs by inhibiting the activity of FXR (39). In this regard, another study showed that the biological link of gut microbiota-GUDCA-FXR was related to glucose intolerance (40). Furthermore, LPCs, the active metabolites of SFA16:0, promoted insulin resistance and cell death in diabetes by activating endoplasmic reticulum (ER) stress (41, 42). In this regard, our study found that high SFA16:0 enhanced the risk association of copresence of low DCA/GUDCA and *CDKAL1* genetic variants with the risk of GDM. Thus, it is also possible that impaired beta-cell function cannot produce enough insulin to cope with increased insulin resistance as caused by high LPC18:0, thereby triggering a high risk of GDM. Hence, the markedly increased susceptibility to GDM due to exposure to both *CDKAL1* rs7747752 C allele and low GUDCA/DCA is biologically plausible although we are not sure whether impaired insulin secretion or increased insulin resistance plays a dominant role. Indeed, molecular mechanisms underlying the interactions between rs7747752 and low GUDCA/DCA for GDM warrant further investigations.

Our findings have public health implications. GDM begets adverse short- and long-term health risks to the mother, developing fetus, and their offspring. Thus, understanding the pathophysiology of GDM and identifying potentially modifiable risk factors and biomarkers for early diagnosis of GDM is essential. In the contention, our study corroborated the link of the *CDKAL1* rs7747752 with GDM in Chinese pregnant women and confirmed that the additive interactions between rs7747752 and low serum levels of GUDCA/DCA for markedly increased risk of GDM, were independent of serum levels of SFA16:0. Notably, the additive interaction effect between rs7747752 and low DCA on the risk of GDM was partly mediated *via* increasing levels of LPC18:0. These risk genetic and metabolomics markers may be useful in the identification of women at high risk of GDM in early pregnancy.

Our research has several limitations. First, our study was a case-control study nested in a population-based prospective cohort of pregnant women in Tianjin, China. Our findings need to be replicated in other Chinese and non-Chinese cohorts of pregnant women. Second, due to busy clinical settings and a limited budget, lifestyle factors such as dietary habits were not collected. Third, we used a two-step GDM screening procedure to identify GDM and some GDM cases might have been missed. Fourth, universal OGTT in early pregnancy was not conducted due to tight budget and we cannot exclude the possibility that some women with GDM may have had prepregnancy impaired glucose tolerance.

In conclusion, we found that *CDKAL1* rs7747752 and serum GUDCA ≤ 0.07 nmol/mL and DCA ≤ 0.28 nmol/mL had significant interactive effects on the risk of GDM in Chinese pregnant women, independent of serum levels of SFA16:0. The additive interaction effect between *CDKAL1* rs7747752 and low DCA on GDM was partially mediated *via* increasing levels of LPC18:0. The discovery of the interactions between *CDKAL1* rs7747752 C risk allele and low DCA/GUDCA is an important step towards precision prevention of GDM in early pregnancy. The additive interaction has potential values in the prediction of GDM in early pregnancy that may benefit from specific intervention if validation studies can confirm our findings in other cohorts of pregnant women populations. It is also warranted to investigate the molecular roles of *CDKAL1* gene and methylthiotransferase in the regulation of metabolic and signaling pathways of BAs, SFAs, and LPCs for increased risk of GDM for better understanding of the etiology of GDM.

## Data Availability Statement

The data and code used during the current study are available from the corresponding author on reasonable request.

## Ethics Statement

The studies involving human participants were reviewed and approved by Ethics Committee of Tianjin Women and Children’s Health Center (TWCHC). The patients/participants provided their written informed consent to participate in this study.

## Author Contributions

XYang, YW and ZF conceived the idea and designed the study. JLeng and WL collected the clinical data. ZF measured the metabolomics and integrated metabolomics data. YW measured the genetics and integrated the genetic data. HW and JLi analyzed the data and wrote the first draft. All the authors gave critical comments and edited the manuscript. XYang and HW took full responsibility for the work, including the study design, access to the data, and decision to submit. All authors contributed to the article and approved the submitted version.

## Funding

This work was supported by the National Natural Science Foundation of China (Grant No: 81870549), the National Key Research and Development Program of China (Grant No: 2019YFA0802300); the Engaged Talents of Guangdong Medical University in 2017 (Grant No: 2XB17028) and the Sailing Plan of Guangdong Province (Grant No: 4YF16001G).

## Conflict of Interest

The authors declare that the research was conducted in the absence of any commercial or financial relationships that could be construed as a potential conflict of interest.

## Publisher’s Note

All claims expressed in this article are solely those of the authors and do not necessarily represent those of their affiliated organizations, or those of the publisher, the editors and the reviewers. Any product that may be evaluated in this article, or claim that may be made by its manufacturer, is not guaranteed or endorsed by the publisher.
