# Supplementary material for: The CDKAL1 rs7747752-Bile Acids Interaction Increased Risk of Gestational Diabetes Mellitus: A Nested Case-Control Study
Source: Front Endocrinol (Lausanne). 2022 Mar 10;13:808956. doi: 10.3389/fendo.2022.808956 (PMC8960111; doi:10.3389/fendo.2022.808956)
Supplement: Supplementary file 1 [file DataSheet_1.docx]

Supplementary Material

# Supplementary Tables

**Supplementary Table 1**. Serum levels of bile acids between different rs7747752 genotypes.

| Characteristic | rs7747752 GG genotypes (n=107) | rs7747752 CG genotypes (n=204) | rs7747752 CC genotypes (n=103) | *P* value |
| --- | --- | --- | --- | --- |
| GUDCA, nmol/mL | 0.04±0.07 | 0.05±0.14 | 0.03±0.03 | 0.150 |
| DCA, nmol/mL | 0.28±0.21 | 0.30±0.30 | 0.29±0.22 | 0.970 |

Abbreviations: GUDCA, glycoursodeoxycholic acid; DCA, deoxycholic acid.

**Supplementary Table 2**. Mediation effects of LPC18:0 and SFA16:0 for interactions between rs7747752 C allele (C vs. G) and low GUDCA, between rs7747752 C allele (C vs. G) and low DCA to increased risk of gestational diabetes mellitus.

|  | Beta (SD) | OR (95% CI) | *P* value |
| --- | --- | --- | --- |
| **Mediation effect of high LPC18:0 for interaction between rs7747752 C allele (C vs. G) and GUDCA to GDM** | | | |
| **Model A (LPC8:0 ≥18.0 nmol/mL as the outcome)** | |  |  |
| rs7747752 C allele & GUDCA ≤ 0.07 nmol/mL | 1.91(1.18) | 6.75(0.67-68.5) | 0.106 |
| **Model B (GDM as the outcome)** |  |  |  |
| LPC8:0 ≥ vs. < 18.0 nmol/mL | 2.89(0.38) | 18.1(8.51-38.3) | <0.001 |
| **Model C (GDM as the outcome)** |  |  |  |
| rs7747752 C allele & GUDCA ≤ 0.07 nmol/mL | 1.97(1.08) | 7.20(0.87-59.4) | 0.067 |
| **Sobel test for mediation effect**^†^ |  |  | 0.114 |
| **Mediation effect of high SFA16:0 for interaction between rs7747752 C allele (C vs. G) and GUDCA to GDM** | | | |
| **Model A (SFA16:0 ≥17.1 nmol/mL as the outcome)** | |  |  |
| rs7747752 C allele & GUDCA ≤ 0.07 nmol/mL | -1.11(1027) | 0.33(0.03-3.97) | 0.383 |
| **Model B (GDM as the outcome)** |  |  |  |
| SFA16:0 ≥ vs. < 17.1 nmol/mL | 0.87(0.24) | 2.39(1.50-3.78) | 0.001 |
| **Model C (GDM as the outcome)** |  |  |  |
| rs7747752 C allele & GUDCA ≤ 0.07 nmol/mL | 2.40(0.90) | 11.1(1.89-64.8) | 0.008 |
| **Sobel test for mediation effect**^†^ |  |  | 0.379 |
| **Mediation effect of high SFA16:0 for interaction between rs7747752 C allele (C vs. G) and DCA to GDM** | | | |
| **Model A (SFA16:0 ≥17.1 nmol/mL as the outcome)** | |  |  |
| rs7747752 C allele & DCA ≤ 0.28 nmol/mL | 0.51(0.53) | 1.67(0.59-4.74) | 0.336 |
| **Model B (GDM as the outcome)** |  |  |  |
| SFA16:0 ≥ vs. < 17.1 nmol/mL | 0.87(0.24) | 2.38(1.50-3.78) | <0.001 |
| **Model C (GDM as the outcome)** |  |  |  |
| rs7747752 C allele & DCA ≤ 0.28 nmol/mL | 1.47(0.44) | 4.36(1.83-10.4) | <0.001 |
| **Sobel test for mediation effect**^†^ |  |  | 0.334 |

Abbreviations: SD, standard definition; OR, odds ratios; CI, confidence intervals; LPC, lysophosphatidylcholines; GUDCA, glycoursodeoxycholic acid; GDM, gestational diabetes mellitus; SFA, saturated fatty acid; DCA, deoxycholic acid.

Model A was adjusted for the variables listed in the adjusted model in table 4.

Model B was adjusted for traditional risk factors in the adjusted model in table 4.

Model C was further adjusted for LPC18:0 ≥ 18.0 nmol/mL or SFA16:0 ≥ 17.1 nmol/mL in addition to the variables listed in the adjusted model in table 4.

^†^ *P* value of Sobel test < 0.05 indicating significant mediation effect.
